# Deficits in functional performance and gait one year after total knee arthroplasty despite improved self-reported function

**DOI:** 10.1007/s00167-016-4234-7

**Published:** 2016-07-19

**Authors:** Josefine E. Naili, Maura D. Iversen, Anna-Clara Esbjörnsson, Margareta Hedström, Michael H. Schwartz, Charlotte K. Häger, Eva W. Broström

**Affiliations:** 1 Department of Women’s and Children’s Health, Karolinska Institutet, Karolinska University Hospital, Stockholm, Sweden; 2 Department of Physical Therapy, Movement & Rehabilitation Sciences, Bouve College of Health Sciences, Northeastern University, Boston, MA USA; 3 Division of Rheumatology, Immunology, and Allergy, Brigham and Women’s Hospital, Harvard Medical School, Boston, MA USA; 4 Department of Clinical Science, Intervention and Technology, Karolinska Institutet, Stockholm, Sweden; 5 Department of Orthopedics, Karolinska University Hospital, Stockholm, Sweden; 6 Gillette Children’s Specialty Healthcare, St Paul, MN USA; 7 Department of Biomedical Engineering, University of Minnesota, Minneapolis, MN USA; 8 Department of Orthopaedic Surgery, University of Minnesota, Minneapolis, MN USA; 9 Department of Community Medicine and Rehabilitation, Physiotherapy, Umeå University, Umeå, Sweden

**Keywords:** Arthroplasty, Knee osteoarthritis, Outcome, Gait, Function, Biomechanics, Patient-reported outcome, Mobile bearing

## Abstract

**Purpose:**

The current literature lacks sufficient information about improvements in gait patterns and function after total knee arthroplasty (TKA) and whether patients return to full function. This study evaluated change in gait, performance-based function, and self-reported function 1 year after TKA in patients with symptomatic knee osteoarthritis and how these aspects interrelate.

**Methods:**

A total of 28 patients (64 % female) with knee osteoarthritis, with a mean age of 66 (±7) years, and 25 age- and gender-matched controls participated in this prospective cohort study. Three-dimensional gait analysis generated comprehensive measures of kinematic and kinetic gait deviations, respectively. Participants completed the Five Times Sit-to-Stand (5STS) test, and the self-reported questionnaire Knee Injury and Osteoarthritis Outcome Score (KOOS), at baseline prior to surgery and 1 year after TKA.

**Results:**

Kinetic gait deviations of both the operated and non-operated limb persisted in patients with knee osteoarthritis at 1 year after surgery, while kinematic gait patterns were comparable to controls. Performance on the 5STS and KOOS scores in patients with knee osteoarthritis improved significantly 1 year after surgery (effect size 0.5–1.5), but did not reach the level of controls. Ten patients with knee osteoarthritis (36 %) exceeded the minimally detectable change on the 5STS.

**Conclusion:**

Measures of overall gait patterns and the 5STS revealed improvements in function 1 year after TKA, but were not restored to the level of healthy controls. Based on change in 5STS performance, we identified patients with substantial improvements in gait patterns. Self-reported measures of function could not detect differences between patients improving in 5STS performance and those who did not. These findings highlight the use of the 5STS in clinical practice since improvement on this test seems to follow the reduction in gait pattern deviations.

**Level of evidence:**

II.

## Introduction

The current literature lacks sufficient information about the degree of functional improvement after total knee arthroplasty (TKA) using objectively measured function including gait patterns [21]. Patients with knee osteoarthritis report decreased pain and improved function following TKA surgery [15, 19], and research indicates patient satisfaction correlates well with both self-reported outcomes and a clinician-based score [2]. Mean improvement in self-reported pain and function is reported to range from 50 to 168 % at 6 months after TKA [35]. At 1 year after TKA, improvements in self-reported pain and function range from 52 to 194 %, where the largest improvements were found in a functional subscale, measuring function in sport and recreation [33]. Self-reported measures of function have been found to be largely influenced by pain, and if perceived pain is greatly reduced, the assessment of function may be overestimated or confused with improved function rather than reduced pain [3].

Despite these improvements, 20–30 % of patients report persistent disability, limited function, reduced quality of life, diminished working capacity, and gait deviations after TKA [24, 38]. Gait pattern in patients operated with unicompartmental knee arthroplasty resembles healthy controls more than patients operated with TKA [37]. In patients with knee osteoarthritis, joint replacements are generally considered successful surgeries. Traditionally, these surgeries were performed on older patients with lower functional demands. Thus, functional improvement has not been considered as important as pain relief. Disability associated with TKA may be conceptualized as a surgical failure since the indication for surgery is pain and impaired function. In clinical practice, the array of available functional outcome measures is usually restricted to self-reported measures and performance-based measures as they do not require special equipment. Detailed knowledge of the relationships between objective measures of function derived from three-dimensional (3D) gait analysis and performance-based function would be advantageous when determining which outcome measures to use in clinical practice.

This study aimed to objectively quantify functional improvements in gait patterns and performance-based function 1 year after TKA in patients with knee osteoarthritis. Further, we aimed to explore the relationships between the degree of improvement in gait pattern, with respect to kinematics and kinetics, in performance-based function using the Five Times Sit-to-Stand (5STS) test, and in self-reported function using a disease-specific questionnaire. In this study, it was hypothesized that (1) patients with knee osteoarthritis would significantly reduce the degree of gait pattern deviation and (2) improve performance on the 5STS 1 year after TKA. Further it was hypothesized that patients with knee osteoarthritis, operated with TKA, would nevertheless not reach the level of healthy controls with regard to gait patterns and performance on the 5STS 1 year after surgery.

## Materials and methods

Forty patients with physician diagnosed primary knee osteoarthritis were included in this prospective cohort study. Patients were recruited from two orthopaedic departments in Stockholm, Sweden (Ortho center, Löwenströmska hospital and Karolinska University Hospital). The inclusion criteria were: being scheduled for TKA within one month; ability to walk 10 m repeatedly without the use of a walking aid; and ability to understand verbal and written information in Swedish. Exclusion criteria were: any previous major orthopaedic surgery in the lower limbs, severe back pain or other lower extremity joint pain, rheumatoid arthritis, diabetes mellitus, neurological disease, BMI >40, and/or other condition affecting walking ability. Twenty-five age- and gender-matched, healthy controls without any known musculoskeletal disease or neurological disorder were recruited through acquaintances between the years 2013 and 2015. The control group was matched to the osteoarthritis group by age strata across five age groups (40–49, 50–59, 60–69, 70–79, and 80–89 years of age). The regional ethical review board in Stockholm, Sweden, approved the study (DNR 2010/1014-31/1). All participants provided informed consent in accordance with the Declaration of Helsinki.

**Fig. 1:**
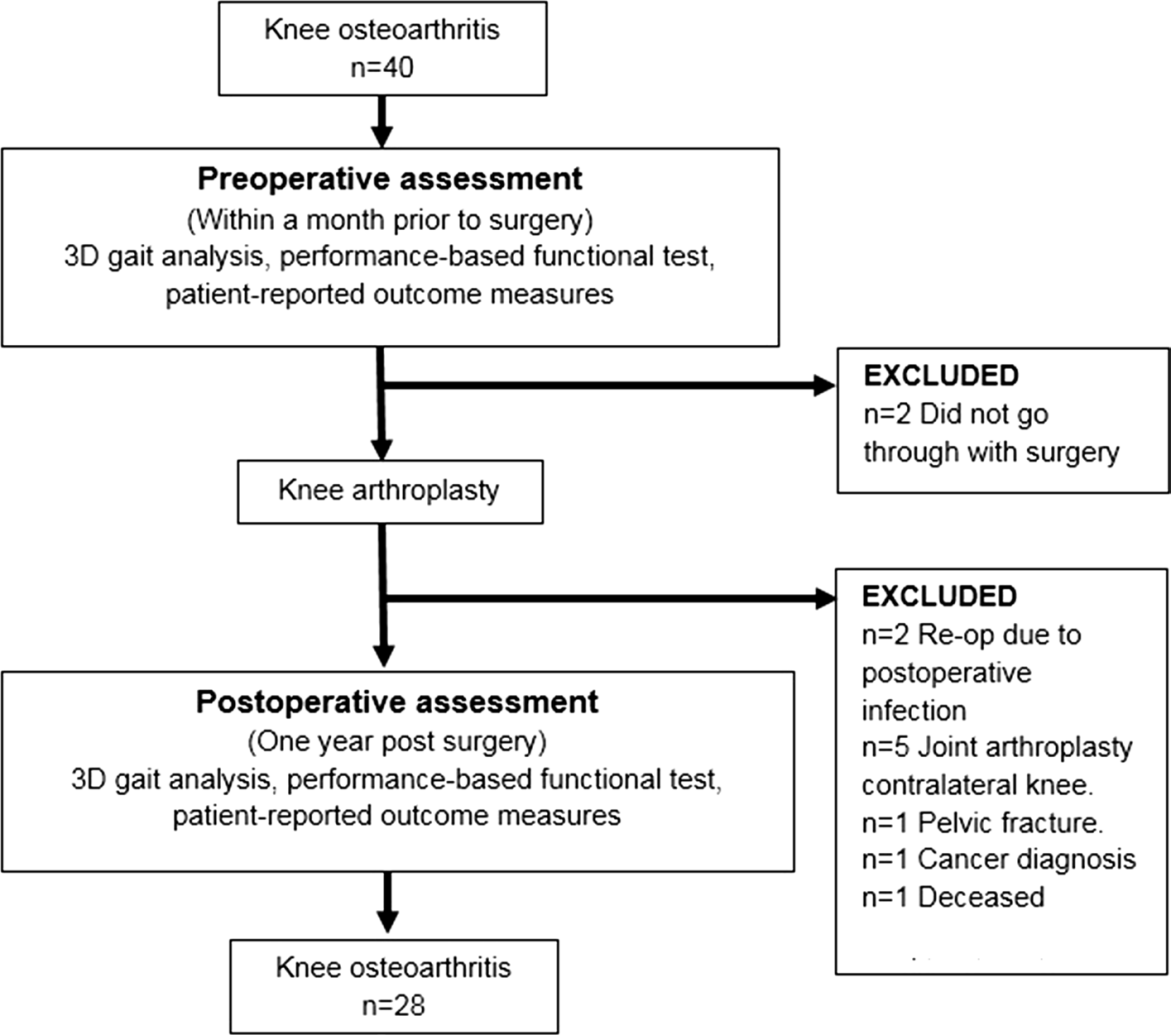
Flowchart of included patients with knee osteoarthritis (OA), test procedures, excluded patients, and patients completing the 1-year follow-up

Twenty-eight patients with knee osteoarthritis, with a mean age of 66 (±7) years, 64 % female, completed the 1-year follow-up (Table 1). The most common reasons for not completing the 1-year follow-up were TKA in the contralateral limb within the subsequent year (*n*=5), not undergoing the planned surgery (*n*=2), or post-operative infection causing re-operation (*n*=2) (Fig. 1). Patients with knee osteoarthritis who did not complete the 1-year follow-up (*n*=12) did not differ statistically from the studied TKA group with respect to the distribution of age, gender, weight, height, BMI, or duration of years with symptomatic knee osteoarthritis (which was evaluated using independent samples *t* tests or the Mann Whitney *U* test, depending on the distribution of data, and a Fishers exact test).

**Table 1:** Baseline characteristics and clinical features of patients with knee osteoarthritis scheduled for knee arthroplasty and in the control group

	Knee OA (*n*=28)	Control group (*n*=25)	Differences between groups
*Characteristics*			*p* value
Mean age, years (SD)	65.7 (7.3)	65.7 (9.5)	n.s.
40–49 years, *n* (%)	0	1 (4)	
50–59 years, *n* (%)	7 (25)	5 (20)	
60–69 years, *n* (%)	12 (43)	9 (36)	
70–79 years, *n* (%)	8 (29)	9 (36)	
80–89 years, *n* (%)	1 (4)	1 (4)	
Female, *n* (%)	18 (64)	16 (64)	n.s.
Body mass index (kg/m^2^), mean (SD)	29.6 (4.6)	24.9 (2.9)	0.003
Body weight (kg), mean (SD)	83.7 (12.8)	72.8 (12.2)	0.000
Height (cm), mean (SD)	168 (8)	171 (8)	n.s.
Symptom duration (years), mean (SD)	7.8 (7.7)	NA	
Previous minor orthopaedic surgery, *n* (%)	14 (50)	NA	
*Modified KL score (1–4b)*			
1–2	–	NA	
3a, *n* (%)	1 (4)	NA	
3b, *n* (%)	4 (14)	NA	
4a, *n* (%)	7 (25)	NA	
4b, *n* (%)	16 (57)	NA	
*Use of analgesics*			
Daily use, *n* (%)	9 (32)	NA	
If necessary (when needed), *n* (%)	11 (39)	NA	
Never (rarely), *n* (%)	8 (29)	NA	

*OA* osteoarthritis; *y* years; *SD* standard deviation; *n* number; *KL* Kellgren and Lawrence; *EQ-5D* EuroQol Five Dimensions; minor orthopaedic surgery refers to knee joint arthroscopy for all but two in the knee OA group which refers to surgical treatment of hallux valgus. Parametric statistics, independent samples *t* tests, were used to calculate differences between OA group and controls. Level of significance set to *p* <0.05

### Protocol

Gait analyses were conducted, and performance-based function was assessed by one of the two experienced physiotherapist (JEN, ACE) at the Motion Analysis Laboratory at Karolinska University Hospital, between the years 2010 and 2015. Each test session started with a physical examination using a standardized protocol. Anthropometric measures were recorded using calibrated scales. Participants started with completing the gait trials, walking barefoot along a 10-m-long pathway at self-selected speed. Recordings were performed in two directions (back and forth). Walking speed, kinematic, and kinetic parameters were collected using an 8-camera motion system (©Vicon Motion Systems Ltd, Oxford, UK) and two force plates (Kistler, Winterthur, Switzerland). Kinetics were expressed as internal moments and total joint power. We used the biomechanical model Plug-In-Gait, where 35 retro-reflective markers were placed on anatomical landmarks [8]. Using this model, good intra-sessional repeatability has been reported [13] and an inter-sessional standard error of 1.8º for global kinematic data in healthy adults [12].

After completing the gait trials, participants completed self-reported measures to allow for a rest period (~20 min), before performing the 5STS [22]. Patients with knee osteoarthritis performed the test protocol twice: once within one month prior to TKA and once again 1 year after surgery. The post-operative evaluation occurred at a median of 12 months (range 11–14 months) following TKA. At the post-operative assessment, patients provided information on their post-operative rehabilitation, duration of rehabilitation, and post-operative complications (e.g. fall accidents, deep vein thrombosis, and wound infections).

### Knee replacement surgery and post-operative rehabilitation

Seven senior orthopaedic surgeons from two different hospitals performed the surgeries. All procedures were performed using a posterior cruciate ligament-retaining cemented TKA.

Post-operative regimes allowed full weight-bearing (together with use of an appropriate walking aid), and rehabilitation was performed according to standard practice at each hospital. Thereafter, rehabilitation was provided in a primary care setting of the patients’ choice. The standard post-operative rehabilitation lasted for a median duration of 3 months (range 1–6 months) after TKA.

### Measures of self-reported function and pain

All study participants completed the Knee Injury and Osteoarthritis Outcome Score (KOOS) [29], which is considered a reliable measure of baseline function and change over time in patients with knee osteoarthritis [7, 34]. The KOOS is divided into five subscales: symptoms, pain, function in activities of daily living (ADL), function in sport and recreation, and knee-related quality of life. All subscales demonstrate adequate test–retest reliability (intra class correlation (ICC) range 0.85–0.9) [7]. Each subscale generates a final score ranging from 0 to 100 where 0 represents “worst” and 100 “best” [29].

Participants also completed a health-related quality of life (HRQoL) questionnaire, the EuroQol five dimensions (EQ-5D) [4], which is considered valid and responsive in patients with chronic pain, including knee osteoarthritis [28]. In the present study, we used the EQ-5D version with three response options for each item (EQ-5D-3L), and the UK EQ-5D value set to obtain a single index value for health state [10].

### Measures of radiographic severity

Pre-operative radiographs were collected according to standard procedures at each hospital. Two experienced orthopaedic surgeons (MH, PG) assessed the radiographs together and provided the radiologic classification of osteoarthritis, according to the modified Kellgren and Lawrence’s classification (KL) ranging from grades I to IV [9]. Radiographs defined as KL scores of 3 to 4 were further subclassified, by incorporating scores of joint space narrowing (JSN) and bone attrition. Thus, a KL grade 3 radiograph with mild JSN was graded 3a and radiographs with more severe JSN 3b. A KL grade 4 radiograph demonstrating complete loss of joint space was divided into 4a if there was no bone attrition and 4b if subchondral bone attrition existed [9].

### Objective measures of gait patterns and function

Three-dimensional gait analyses were performed, rendering measures of overall gait pattern deviations, using the Gait Deviation Index for kinematics (GDI) and kinetics (GDI-kinetic). The GDI and GDI-kinetic allow comparison of an individual’s gait pattern against the gait of a reference group (*n*=59 for GDI, *n*=56 for GDI-kinetic) [11, 30, 32]. Reference subjects were selected from the control database at the Motion Analysis Laboratory. The GDI is calculated from the pelvis and hip kinematics in all three anatomical planes, the knee and ankle in the sagittal plane and foot progression in the transversal plane [32]. The GDI-kinetic is calculated from the hip, knee, and ankle moments in the frontal and sagittal plane and total joint power in the hip, knee, and ankle [30]. Each limb is considered independently. A GDI or GDI-kinetic score of ≥100 represents normal gait pattern, whereas each ten-point decrement below 100 represents one standard deviation from normal gait and indicates deviation in kinematics (GDI) or kinetics (GDI-kinetic) in one or more joints. In patients with rheumatoid arthritis, ICC of the GDI is 0.952 and standard error of measurement (SEM) 1.92 scores [11]. Five gait trials, with clean force plate strikes, were analysed for each participant, at each test session (pre- and post-operative). The GDI and GDI-kinetic were averaged for these trials to obtain one value for each limb (operated, non-operated) for each index. Walking speed was normalized by gravity and leg length, as described by Hof [16]. Calculations of 3D gait analysis data were performed using the software program MATLAB^®^, R2014a (The MathWorks, Inc, Natick, MA).

The Five Times Sit-to-Stand (5STS) test is a timed test where the participant is asked to rise from a seated position to a standing position five times as quickly as possible [22]. Performance on this test is associated with quadriceps strength and functional performance up to 6 months after TKA in patients with knee osteoarthritis [6]. The test shows excellent relative and absolute reliability in older adults (ICC 0.95, SEM 0.9 s) [14]. The test was performed twice, timed to a hundredth of a second, and the best (lowest) value was used in the analysis. To determine post-operative improvement in objectively measured function, the 5STS was used as it is a method easily applicable in clinical practice. Patients with knee osteoarthritis were grouped and compared based on their 5STS results according to the established minimally detectable change (MDC) of 2.5 s in 5STS performance [14]. Patients with a reduction in time equal to or greater than 2.5 s were considered to have a “Good 5STS outcome”, and those with a reduction of less than 2.5 s or an increase in time were considered to have a “Bad 5STS outcome”.

### Statistical analysis

Statistical analyses were performed using IBM SPSS Statistics version 22. A significance level was set at *p* <0.05. To assess change in function pre- and post-operatively, paired sample *t* tests or Wilcoxon signed-rank tests were used, depending on the distribution of data. Normal distribution of the data was assessed using Shapiro–Wilk’s test and Q–Q plots. To evaluate the magnitude of change in function, effect sizes (Cohen’s *d*) were calculated along with 95 % confidence intervals (CI) [26]. A Fishers exact test was used to determine whether the proportion of improvement on the 5STS was beyond the MDC, and change in GDI and GDI-kinetic was statistically different [22]. Groups were defined according to the MDC of each specific measurement, change in time by 2.5 s on the 5STS [14] and change by 5.4 units in GDI and GDI-kinetic scores, respectively [11].

To evaluate differences in function between patients with TKA and controls, independent samples *t* tests or the Mann Whitney *U* test was used, depending on data distribution. To determine the sample size needed to detect a difference of 5 GDI units between patients with knee osteoarthritis and healthy controls, and with the power set at 0.8, a sample size of 24 subjects was required in the knee osteoarthritis group. Since the control group consisted of a functionally symmetric population, we arbitrarily chose the right leg in the statistical analysis.

## Results

### Improvements in objective measures of function

Overall gait pattern deviations decreased significantly, indicated by an increase in GDI scores on the operated limb, GDI-kinetic scores on the operated and the non-operated limb (Table 2). Compared to the control group, patients with TKA demonstrated significantly lower GDI-kinetic scores and reduced walking speed after surgery, while kinematic GDI scores were comparable to the controls. Overall performance on the 5STS improved after surgery and was still significantly lower than the control group value (Table 2).

**Table 2:** Functional assessment at baseline prior to knee arthroplasty and at 1-year follow-up in patients with knee osteoarthritis and controls

	TKA (*n*=28)				Controls (*n*=25)
	Pre-TKA baseline mean (SD)	Mean change (SD)	Effect size (95 % CI)	Post-TKA mean (SD)	Control group mean (SD)
*Gait pattern*					
GDI operated	87.2 (10.5)	5.8 (13)*	0.4 (0.1–0.8)	93 (9.9)	96.6 (9)
GDI non-operated	89.3 (12.6)	4.5 (13.8)	0.3 (−0.1–0.7)	93.9 (8.7)	96.6 (9)
GDI-kinetic operated	90.1 (6.9)	3.8 (6.9)*	0.5 (0.1–0.9)	93.8 (8.4)	100 (8.6)^□^
GDI-kinetic non-operated	87.9 (8.3)	6.7 (7.8)^‡^	0.9 (0.4–1.3)	94.7 (8.6)	100 (8.6)^□^
Walking speed (m/s)	1.11 (0.20)	0.07 (0.15)*	0.5 (0.1–0.8)	1.17 (0.18)	1.30 (0.18)*
ND walking speed	0.37 (0.07)	0.02 (0.05)*	0.5 (0.1–0.9)	0.40 (0.06)	0.44 (0.06)*
*Self-reported function*					
KOOS (0–100)					
Pain	45.3 (15.2)	32.7 (22)^‡^	1.5 (0.9–2.0)	78 (20.6)	96.9 (5.3)^‡^
Symptoms	41.2 (20.5)	34.6 (23.1)^‡^	1.5 (1.0–2-0)	75.9 (18.3)	94.9 (6.7)^‡^
ADL	57.2 (15.3)	23.8 (17.9)^‡^	0.8 (0.4–1.2)	81.1 (19.1)	96.3 (6.3)^‡^
Sport/Rec	22 (21.8)	17.1 (28.3)^□^	0.6 (0.2–1.0)	39.1 (27)	89.6 (14.9)^‡^
Knee-related QoL	28.1 (12)	33.6 (23.5)^‡^	1.4 (0.9–2.0)	61.6 (26.8)	90.5 (13.8)^‡^
EQ-5D					
Score (−0.594–1)	0.61 (0.22)	0.14 (0.27)^□^	0.5 (0.1–0.9)	0.75 (0.26)	0.93 (0.1)^‡^
*Performance-based function*					
5STS, s	14.5 (4.2)	−1.6 (4.4)^□^	0.6 (0.2–1.0)	12.8 (4.8)	9.9 (2.9)^□^

*TKA* total knee arthroplasty; *SD* standard deviation; *CI* confidence interval; *n* number; *GDI* Gait Deviation Index; *ND* non-dimensionalized; *KOOS* Knee Injury and Osteoarthritis Outcome Score; *ADL* activities of daily living; *Sport/Rec* sport and recreation; *QoL* quality of life, *EQ-5D* EuroQol 5 Dimensions; *5STS* Five Times Sit-to-Stand. Parametric and nonparametric statistics were used; paired samples *t* test and Wilcoxon Sign rank test to compare pre- and post-operative results within the TKA group; independent samples *t* test and Mann Whitney *U* test to calculate differences between TKA group post-operatively and controls, which are reported in the column for control group mean. Level of significance set to * *p* < 0.05, ^□^ *p* < 0.01, ^‡^ *p* < 0.001

### Functional improvement represented by performance on the 5STS

**Fig. 2:**
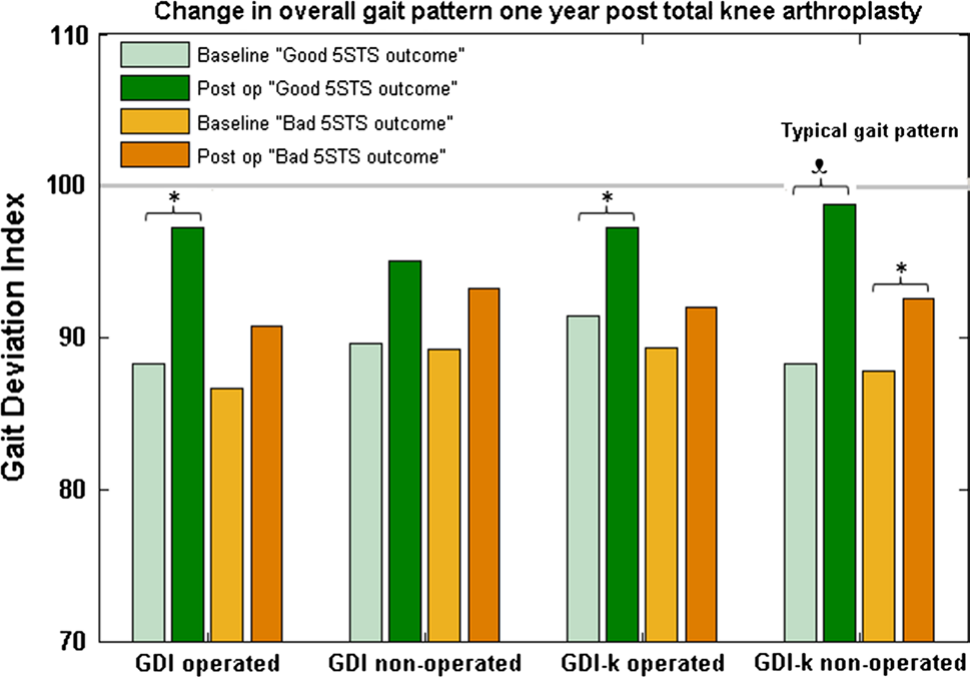
Overall gait pattern, quantified using the Gait Deviation Index for kinematics (GDI) and kinetics (GDI-k), at baseline and 1 year after total knee arthroplasty in patients with knee osteoarthritis. Patients were grouped by change in performance on the Five Times Sit-to-Stand test. Level of significance set to * *p*  < 0.05, □ *p* < 0.01

Patients with TKA were dichotomized into two groups based on the MDC of 2.5 s on the 5STS: the “Good 5STS outcome” (*n*=10, 36 %) and the “Bad 5STS outcome” group (*n*=18, 64 %). Significant improvements were found in GDI and GDI-kinetic scores in the “Good 5STS outcome” group (Fig. 2). The “Good 5STS outcome” group demonstrated improvements in GDI scores on the operated limb and GDI-kinetic scores on both the operated and non-operated limb, respectively. In the “Bad 5STS outcome” group, no significant changes were found in GDI scores, while GDI-kinetic scores on non-operated limb increased significantly (Fig. 2).

### Improvements in self-reported measures of function and pain

**Fig. 3:**
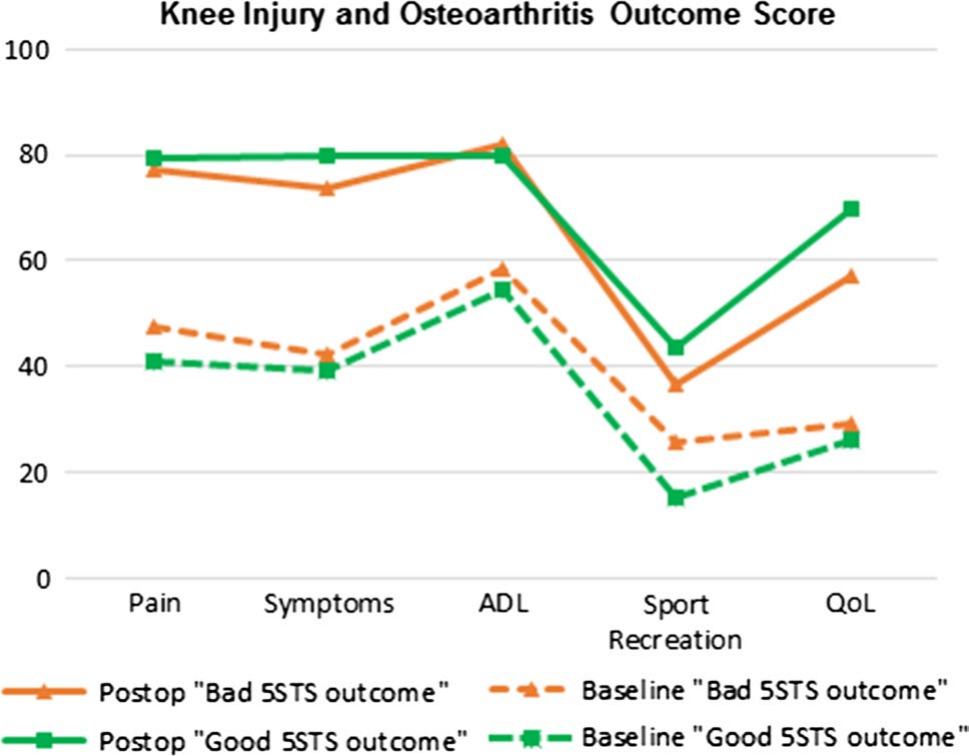
Patient-reported function, evaluated using the Knee Injury and Osteoarthritis Outcome Score (KOOS), at baseline and 1 year after total knee arthroplasty in patients with knee osteoarthritis. Patients were grouped by change in performance on the Five Times Sit-to-Stand test. ADL, activities of daily living; QoL, knee-related quality of life

Data from the KOOS and EQ-5D indicated improvement in all areas, pain, symptoms, function in ADL, function in sport and recreation, knee-related quality of life, and HRQoL (Table 2). The largest functional improvements were found in KOOS subscales “pain” (73 %), “symptoms” (85 %) and “knee-related quality of life” (121 %). Compared to controls, lower scores were found in all subscales of KOOS and in EQ-5D (Table 2). When comparing KOOS subscale scores between the “Good” and “Bad” 5STS outcome groups among patients with TKA, no significant differences were found in either subscale (Fig. 3).

## Discussion

The most important finding of the present study was that objectively measured function, represented by gait patterns and the 5STS, improved significantly, but did not reach the level of healthy controls. Based on change in 5STS performance, we identified patients with substantial improvements in gait patterns, while self-reported measures of function could not differentiate between patients improving in functional performance and those who did not.

Pre-operatively, lower GDI-kinetic scores were observed in the contralateral limb compared to the soon-to-be-operated limb. After surgery, the largest gait pattern improvements were found in GDI-kinetic scores of the contralateral (non-operated) limb. These results suggest TKA has a positive impact on gait patterns for both limbs, although deviations are still present 1 year post-operatively as compared to healthy controls. These results corroborate the findings of Metcalfe *et al.* [23] that gait pattern recovery at the contralateral knee is variable and often incomplete. Using a 1-year follow-up of gait patterns may be a too short period of time with regard to restoring joint loading. It has been suggested that functional recovery continues beyond 1 year and even up to 2 years after surgery [20].

We hypothesized that performance on the 5STS would improve after surgery, and in this sample the performance improved significantly. Nevertheless, the improvement only exceeded the MDC in 10 (36 %) of the 28 TKA patients, while the rest remained unchanged. Patients classified as having a “Good 5STS outcome” displayed significant improvements in GDI and GDI-kinetic scores, whereas patients classified as having a “Bad 5STS outcome” did not. Performance on this test is associated with quadriceps strength, and this test is considered a surrogate measure for lower limb strength [22, 27, 31]. Studies also report factors such as balance, age, weight, and sensorimotor competence play an important role in 5STS performance [22, 31]. Christiansen *et al.* [6] evaluated weight-bearing asymmetry during the 5STS in patients with knee osteoarthritis and concluded that greater amounts of weight-bearing asymmetry correlated with poorer functional performance up to 6 months after TKA. Alnahdi *et al.* [1] found patients with TKA displayed unloading of the operated limb, shifting the load to the contralateral limb when performing a sit-to-stand test 1 year after surgery.

Comparing self-reported function and pain between the “Good 5STS outcome” and “Bad 5STS outcome” groups, we found no differences, suggesting that self-reported measures are not able to detect change in performance on this test. Results of our study are consistent with prior studies demonstrating that improvements in self-reported measures are greater than improvement in performance-based function [17, 25, 35]. Boonstra *et al*. [3] suggest self-reported measures of function are highly influenced by pain. After a successful TKA, pain is substantially reduced and this pain relief may lead patients to overestimate self-reported function or confuse improvements in function with improvements in pain [3].

Limitations of the present study include that our sample consisted of individuals without comorbidities who were able to ambulate without the use of a walking aid. Consequently, we cannot generalize the results to all patients with knee osteoarthritis. The sample size is small, yet consistent with, and even larger than other studies using 3D gait analysis [18, 23, 36]. However, these patients presented with significantly higher weight and BMI compared to the control group, and this difference in BMI may have influenced gait patterns and 5STS performance [31]. We recognize that the use of seven different senior consultant orthopaedic surgeons to perform the surgeries increases the generalizability of the results, as this is representative of surgical practice. However, individual capability of an orthopaedic surgeon could be argued to influence the outcome of the surgery. Examining the inter-surgeon effect was beyond the scope of the present study. Additionally, there was no standardization of the post-operative rehabilitation following TKA to control the impact of rehabilitation on gait pattern or 5STS performance. Anecdotally, the standard protocol for rehabilitation following TKA did not differ between the two orthopaedic departments in this study. The rehabilitation programme included inpatient physiotherapy (>1 week), followed by physiotherapy in a primary care setting of the patient’s choice for varying lengths of time. Median time for post-operative rehabilitation in this sample was 3 months. The MDC of the 5STS is based on a sample of 29 females, mean aged 73.6 years who were able to walk at least 10 m and stand at least 10 min without an assistive device and who had no neurological disease, diabetes, visual deficits, or amputated extremities [14]. Caution should be taken not to overestimate the importance of this value, since the two studied groups (ours and Goldberg’s) might differ substantially. Additionally, we used the MDC reported for GDI [10], to evaluate change in GDI-kinetic scores. Since the GDI-kinetic is an analogue of the GDI and the magnitude of change of the two measures was similar, the MDC of 5.4 units was considered to be useful in the evaluation of GDI-kinetic.

Based on our findings, we advocate for use of the 5STS in clinical practice, as improvement beyond the MDC in 5STS performance appears to be accompanied by significant reductions in kinematic and kinetic gait pattern deviations. Additionally, our data indicate self-reported measures of function have limited use in detecting change in performance-based function. This has implications for pre-operative patient education. Specifically, we believe it is important to inform patients undergoing surgery that TKA is an effective treatment for alleviating pain and symptoms of the affected joint, but activities of daily living and more strenuous activities requiring power, strength, and balance may not become fully restored after surgery. Future research should evaluate the impact of specific exercise programmes on restoring normal joint loading patterns and functional performance. Additionally, we recommend studies to examine whether more extensive post-operative rehabilitation could lead to greater gains in performance-based function and in reductions in kinetic gait deviations. It would also be of interest to evaluate whether joint loading deviations persist at two or more years after surgery.

## Conclusion

Measures of overall gait patterns and the 5STS revealed improvements in function 1 year after TKA, but were not restored to the level of healthy controls. Based on change in 5STS performance, we identified patients with substantial improvements in gait patterns. Self-reported measures of function could not detect differences between patients improving in 5STS performance and those who did not. These findings highlight the use of the 5STS in clinical practice since improvement on this test seems to follow the reduction in gait pattern deviations.
